# Editorial: Biomimetics and immuno-informed biomaterials: a functional role in immune response and *in vivo* reprogramming

**DOI:** 10.3389/fimmu.2025.1595675

**Published:** 2025-06-05

**Authors:** Santanu Kaity, Manjari Singh, Prem Kumar Govindappa, Manash K. Paul, Subhadeep Roy

**Affiliations:** ^1^ Department of Pharmaceutics, National Institute of Pharmaceutical Education and Research, Kolkata, India; ^2^ Department of Pharmaceutical Sciences, Assam University, Silchar, India; ^3^ Department of Orthopaedics ans Sports Medicine, University of Arizona College of Medicine, Tucson, AZ, United States; ^4^ Department of Pulmonary and Critical Care Medicine, David Geffen School of Medicine, University of California Los Angeles, Los Angeles, CA, United States; ^5^ Department of Pharmacology and Toxicology, National Institute of Pharmaceutical Education and Research, Kolkata, India

**Keywords:** biomimetics, biomaterials, immune response, *in vivo* reprogramming, functional biomaterial

Smart biomaterials with comprehensive customization to fulfill difficult tissue engineering, regenerative medicine, and drug delivery needs have driven biomedical research growth. Medical absorption of biomaterials as cargo or therapeutic entities is still hindered by the host immune response. Biomimetics and immune-informed biomaterials use biomimicry, which our immune system admires. Immune-informed biomaterials are made by surface modification or immune-modulating agent integration to bypass the immune response-based defensive system. Thus, the smart material-based bionic environment speeds up cell proliferation and tissue repair without causing inflammation. Microenvironmental reprogramming of the application site can be easy and improve patient response to treatment. Smart biomaterials modulate immune responses by boosting positive responses, dampening foreign body reactions, or recruiting macrophage-like immune cells to assist tissue regeneration. Advanced methods include cell membrane-camouflaged nanoparticles, nanoparticles containing immune-modulating chemicals, and surface-modified hydrogels, and these are researched for tissue engineering and biomedical applications. Silk, extracellular matrices, and natural polymers were also substantially modified to minimize material-triggered immune responses and facilitate *in vivo* reprogramming.

Moulton et al. presented an article on the topic “Navigating the nano-bio immune interface: advancements and challenges in CNS nanotherapeutics”. The authors seek to examine the ability of nanoparticles (NPs) to alter immune responses in the central nervous system (CNS), with the goal of developing feasible nanotherapeutic techniques for neurological diseases. Nanoparticles are being studied as vehicles for immunomodulatory medicines that target specific regions of the central nervous system. They are used to deliver nucleic acid-based treatments for gene expression regulation and immune response modulation. Research demonstrates NP-mediated immune modulation in neurological illnesses such as multiple sclerosis, stroke, Alzheimer’s disease, and Parkinson’s disease. The role of the nano-bio interface, specifically the formation of the biomolecular corona, in influencing nanoparticle behavior and immune recognition within the central nervous system is also investigated. The goal is to guide the development of focused, safe, and effective nanotherapeutic approaches for various CNS disorders, with the potential to alter treatment methodologies and improve patient outcomes.

Gou et al. published a study report titled “Macrophages in guided bone regeneration: potential roles and future directions”. The authors have investigated the role of macrophages in guided bone regeneration (GBR) and the formation of GBR membranes in order to affect immune responses for improved bone healing. The key objectives are as follows: 1) investigate macrophages in the context of bone defect repair for their function in osteogenesis, fibrous tissue formation, membrane disintegration, and fibrous encapsulation; 2) investigate the effect of various GBR membranes on macrophage recruitment and polarization, which affects the immune response and bone regeneration outcomes; 3) create GBR membranes that stimulate macrophage recruitment and control their polarization to prioritize bone regeneration over inflammation or fibrous tissue formation; 4) identify and propose solutions to challenges such as developing sophisticated delivery systems for macrophage activation agents, reducing interference from bone graft materials and dental implants, and better understanding the relationships between membrane degradation, macrophage responses, and effective bone regeneration.

Schoberleitner et al. published a study titled “Silicone implant surface microtopography modulates inflammation and tissue repair in capsular fibrosis”. The authors have investigated the effect of silicone mammary implant (SMI) surface roughness on immune responses and capsular fibrosis. The key objectives are as follows: assessments of the effect of various levels of SMI surface roughness on acute inflammatory reactions, fibrinogen deposition, and progression of the fibrotic cascade.

Research suggests that reducing surface roughness to 4 μm can improve immune response, wound healing, and reduce fibrosis. There is merit to examining the specific proteins that bind to textured implant surfaces to better understand their roles as potential mediators in pro-inflammatory and pro-fibrotic pathways. Analyzing the implant capsule composition, specifically the expression of intracapsular Heat Shock Protein 60 (HSP60), is key to better understanding the complex interactions between stress responses and immune activation that determine long-term tissue outcomes. These aims are based on a study of 10 patients, with an emphasis on intra- and inter-individual assessments to provide comprehensive insights into the relationship between SMI surface features and host immune responses.

Tripathi et al. have published an article titled “Material Matters: Exploring the Interplay between Natural Biomaterials and the Host Immune System”. The authors seek to investigate the complex interactions between natural biomaterials and the immune system in order to improve the design and effectiveness of medical implants and devices. The key objectives are as follows: to 1) investigate the methods by which the immune system identifies native biomaterials as foreign molecules, activating immune cells such as macrophages, dendritic cells, and T cells; 2) analyze the sequence of events following immunological activation, including the secretion of pro-inflammatory cytokines and chemokines, and determining whether these responses are beneficial or damaging depending on the type of biomaterial and the degree of the immune reaction; 3) investigate the effect of specific biomaterial surface properties, such as charge and hydrophobicity, on immune cell activity, notably activation and differentiation; 4) create and develop biomaterials that contain immunomodulatory chemicals, such as anti-inflammatory cytokines, in order to foster a tolerogenic environment and reduce the likelihood of rejection; 5) using knowledge gained from the interaction of biomaterials and the immune system to create medical devices and implants that increase positive immune responses, hence improving therapeutic outcomes and decreasing negative reactions.

Ghosh et al. published a research study titled “Piezoelectric-based bioactive zinc oxide-cellulose acetate electrospun mats for efficient wound healing: an *in vitro* insight”. The authors have proposed the development and testing of a bioactive wound dressing that actively participates in the healing process using piezoelectric properties. The key objectives are as follows: to 1) fabricate electrospun nanofibrous mats from cellulose acetate (CA) loaded with zinc oxide (ZnO) nanoparticles to take advantage of piezoelectric capabilities for wound healing—the structural and functional characteristics of the produced mats were evaluated using methodologies such as Scanning Electron Microscopy (SEM), Fourier Transform Infrared Spectroscopy (FTIR), Thermogravimetric Analysis (TGA), mechanical testing, degradation analysis, porosity measurement, haemolysis assay, and piezoelectric d33 coefficient measurement; 2) investigate the effect of incorporating ZnO nanoparticles into CA fibres on the piezoelectric coefficient of nanofibrous mats; 3) perform cell culture experiments to investigate cell adhesion, proliferation, and migration on nanofibrous mats, in order to assess their ability to improve wound healing; 4) verify that ZnO-infused CA nanofibrous mats may greatly improve wound healing, making them a feasible therapeutic treatment alternative.

Focused research on biomimetic materials can address complex tissue engineering, *in vivo* reprogramming, and bioactive delivery needs by manipulating the body’s defense system.

This thematic Research Topic reviews and publishes cutting-edge research on biomimetic and immuno-informed biomaterials for immune-response regulation and *in vivo* reprogramming. This Research Topic showcases significant advancements in this biomedical research segment and its future prospects. This Research Topic should encourage researchers in this section to develop novel biomaterials that can improve human health and quality of life.

